# Vertical Transmission of SARS-CoV-2: A Systematic Review of Systematic Reviews

**DOI:** 10.3390/v13091877

**Published:** 2021-09-20

**Authors:** Salihu S. Musa, Umar M. Bello, Shi Zhao, Zainab U. Abdullahi, Muhammad A. Lawan, Daihai He

**Affiliations:** 1 Department of Applied Mathematics, Hong Kong Polytechnic University, Hong Kong, China; salihu-sabiu.musa@connect.polyu.hk; 2 Department of Mathematics, Kano University of Science and Technology, Wudil 713101, Nigeria; mukhazah3@gmail.com; 3 Centre for Eye and Vision Research (CEVR) Limited, Hong Kong Science Park, Hong Kong, China; umar.m.bello@connect.polyu.hk; 4 Department of Physiotherapy, Yobe State University Teaching Hospital, Damaturu 620101, Nigeria; 5 JC School of Public Health and Primary Care, Chinese University of Hong Kong, Hong Kong, China; zhaoshi.cmsa@gmail.com; 6 CUHK Shenzhen Research Institute, Chinese University of Hong Kong, Shenzhen 518000, China; 7 Department of Biological Sciences, Federal University Dutsinma, Katsina 821101, Nigeria; zainabdeedeeumar@gmail.com

**Keywords:** COVID-19, pandemic, vertical transmission, pregnancy, systematic review

## Abstract

The COVID-19 pandemic has hugely impacted global public health and economy. The COVID-19 has also shown potential impacts on maternal perinatal and neonatal outcomes. This systematic review aimed to summarize the evidence from existing systematic reviews about the effects of SARS-CoV-2 infections on maternal perinatal and neonatal outcomes. We searched PubMed, MEDLINE, Embase, and Web of Science in accordance with PRISMA guidelines, from 1 December 2019 to 7 July 2021, for published review studies that included case reports, primary studies, clinical practice guidelines, overviews, case-control studies, and observational studies. Systematic reviews that reported the plausibility of mother-to-child transmission of COVID-19 (also known as vertical transmission), maternal perinatal and neonatal outcomes, and review studies that addressed the effect of SARS-CoV-2 infection during pregnancy were also included. We identified 947 citations, of which 69 studies were included for further analysis. Most (>70%) of the mother-to-child infection was likely due to environmental exposure, although a significant proportion (about 20%) was attributable to potential vertical transmission of SARS-CoV-2. Further results of the review indicated that the mode of delivery of pregnant women infected with SARS-CoV-2 could not increase or decrease the risk of infection for the newborns (outcomes), thereby emphasizing the significance of breastfeeding. The issue of maternal perinatal and neonatal outcomes with SARS-CoV-2 infection continues to worsen during the ongoing COVID-19 pandemic, increasing maternal and neonatal mortality, stillbirth, ruptured ectopic pregnancies, and maternal depression. Based on this study, we observed increasing rates of cesarean delivery from mothers with SARS-CoV-2 infection. We also found that SARS-CoV-2 could be potentially transmitted vertically during the gestation period. However, more data are needed to further investigate and follow-up, especially with reports of newborns infected with SARS-CoV-2, in order to examine a possible long-term adverse effect.

## 1. Introduction

COVID-19, a disease caused by SARS-CoV-2, is a member of the *Coronaviridae* family which is mainly transmitted through air droplets [1,2], but other routes of transmission have been reported [3]. These include maternal perinatal transmission (which could be due to biological or social factors) [4,5,6,7,8,9], vertical transmission (direct transmission between mother-to-child during pregnancy) [10,11,12,13], and environmental transmission [14,15].

Maternal physiological changes during pregnancy predispose pregnant women to infectious diseases [3,4,16,17,18]. Most early systematic reviews that reported mother-to-child transmission during pregnancy highlighted that vertical transmission of SARS-CoV-2 is plausible, even though there was insufficient evidence at the early stage of the pandemic, due to scarcity or inconsistencies in the reported data. Thus, emphasis should be placed towards further investigating and monitoring possible infection in the neonates, born to COVID-19-infected mothers [16,19,20]. Moreover, the studies that reported the possibility of mother-to-child transmission of COVID-19 during pregnancy highlighted the need for additional data to ascertain if the transmission occurs via the transplacental route, transcervical route, or environmental exposure [3,4,5,13,18,21]. It is therefore imperative to investigate the mechanism(s) through which SARS-CoV-2 reaches the fetus, to proffer ways to prevent neonatal infection, optimize pregnancy management, and eventually better understand SARS-CoV-2 epidemiology.

Several reports have shown that vertical transmission of SARS-CoV-2 is possible and likely happens in a minority of cases of maternal SARS-CoV-2 infection, especially during the third trimester of pregnancy [13,20,22,23,24]. The rates of mother-to-child infection are similar to those of other pathogens that cause congenital infections [13,25]. Notwithstanding, given the paucity of early-trimester data, it is challenging to further assess such data regarding mother-to-child transmission in early pregnancy and the potential risk for consequent fetal morbidity and mortality [13]. Thus, this indicates an urgent need to investigate the possibility of SARS-CoV-2 vertical transmission during pregnancy, and to design a strategic public health response to this scenario.

Here, we present a comprehensive systematic review of the existing literature to demonstrate the effect of COVID-19 on transplacental transmission with clinical manifestation in the neonates that resembles neurological signs and symptoms of COVID-19. In particular, this systematic review of systematic reviews intended to report the possibility of vertical transmission related to SARS-CoV-2 during pregnancy. The study consisted of a large collection of systematic reviews that included studies recruiting many pregnant women infected with SARS-CoV-2 and neonatal outcomes. We believe our findings will have significant contributions to the current literature and provide more suggestions for clinical and preventive practice guidelines against SARS-CoV-2 worldwide.

### Objectives of the Study

This study aimed to answer the following questions: (i) Are the current evidence supporting the possibility of vertical transmission of SARS-CoV-2 adequate? (ii) Are there enough evidence to ascertain the possibility of mother-to-child transmission of SARS-CoV-2 during pregnancy?

## 2. Methods

### 2.1. Search Strategy and Study Screening Processes

This study adopted the ‘Preferred Reporting Items for Systematic Reviews and Meta-Analyses’ (PRISMA) guidelines [26]. We conducted a systematic review of systematic reviews of the literature to report an important scenario of mother-to-child transmission of SARS-CoV-2, via vertical transmission route, during pregnancy that covered published peer-reviewed articles from 1 December 2019 to 7 July 2021. A comprehensive literature search was conducted via the following medical databases: PubMed, MEDLINE, Embase, and Web of Science, for papers published in English, on human participants. Search terms used across all databases included: (COVID-19 OR SARS-CoV-2) AND (antenatal OR prenatal OR vertical transmission OR pregnancy OR pregnant OR delivery OR infant OR neonate OR newborn) AND (systematic review). Further, relevant references were manually searched via the reference lists of included studies to ensure robust literature coverage. Citations were independently managed by two authors (S.S.M. and U.M.B.) using EndNote X9.0. All duplicates were removed using the ENDNOTE built-in ‘Find Duplicates’ feature. The two authors (S.S.M. and U.M.B.) independently screened the titles, abstracts, and full text of the non-duplicate generated citations and identified the full texts that met the eligibility criteria of the study.

### 2.2. Study Selection and Eligibility Criteria

Systematic reviews (SR) that assessed vertical transmission of COVID-19 were included in this study if the: (i) SR reported an explicit search strategy in popular databases; (ii) SR examined SARS-CoV-2 vertical transmission during pregnancy; (iii) SR aimed to assess maternal perinatal SARS-CoV-2 infection with neonatal outcomes, and; (iv) SR was published in the English language or with at least an abstract in the English language [26]. To ensure adequate coverage of the literature, we included SRs that reported the possibility of vertical transmission of severe acute respiratory illnesses, which include SARS-CoV-2, Middle East Respiratory Syndrome coronavirus (MERS-CoV), and Severe Acute Respiratory Syndrome coronavirus (SARS-CoV).

Non-SR studies, such as primary studies, clinical practice guidelines, overviews, case reports, and other types of studies aimed at synthesizing evidence of vertical transmission of SARS-CoV-2, were excluded. Studies with irrelevant topics, or lack of relevant information (data), or ineligible article types were also excluded. Furthermore, we excluded studies that reported plausibility of SARS-CoV-2 vertical transmission published in a non-English language. The searching and screening processes of the literature were independently conducted by the two authors (S.S.M. and U.M.B.), and discrepancies were resolved via discussions amongst the authors. 

### 2.3. Quality Assessment

We adopted the modified version of the quality assessment tool for systematic reviews and meta-analyses, entitled “Overview Quality Assessment Questionnaire (OQAQ; Supplementary Materials Table S2) [27], for assessing the methodological quality of the included SRs. The OQAQ tool is flexible for evaluating the quality of studies with multiple study designs. This tool is widely used in public health studies [28,29,30,31,32]. The scoring ranges from 0 to 18 points, with higher scores indicating better methodological quality. SRs scoring 14 points or above are regarded as having higher quality, whereas scores of less than 14 points are rated as low quality [27,28]. Two authors (S.S.M. and U.M.B.) independently assessed methodological quality of the included SRs. Discrepancies in the outcome of the independent quality ratings were resolved via discussions between the authors. For details, see Supplementary Materials Table S3.

### 2.4. Data Extraction and Synthesis

To meet the standard criteria of the review, two authors (S.S.M. and U.M.B.) independently extracted all the relevant data using a pre-designed excel sheet. The data extracted included: authors details; database(s) where SR was located; date of publication; number of included primary studies in the SR; numbers of pregnant women, newborns, infected newborns, maternal deaths, and newborn deaths; mode of delivery, remark on vertical transmission reported in the SR, and; sensitivity analysis in studies with meta-analysis. The data extracted were compared, and any inconsistency in the data was rectified by further deliberations among the authors. Furthermore, the data obtained were synthesized to report issues of SARS-CoV-2 vertical transmission during pregnancy. The data synthesis included all the systematic reviews reports on the incidence of SARS-Cov-2 vertical transmission during pregnancy, i.e., direct mother-to-child transmission of SARS-CoV-2 during pregnancy.

## 3. Results

### 3.1. Literature Search Findings and Study Characteristics

**Figure 1:**
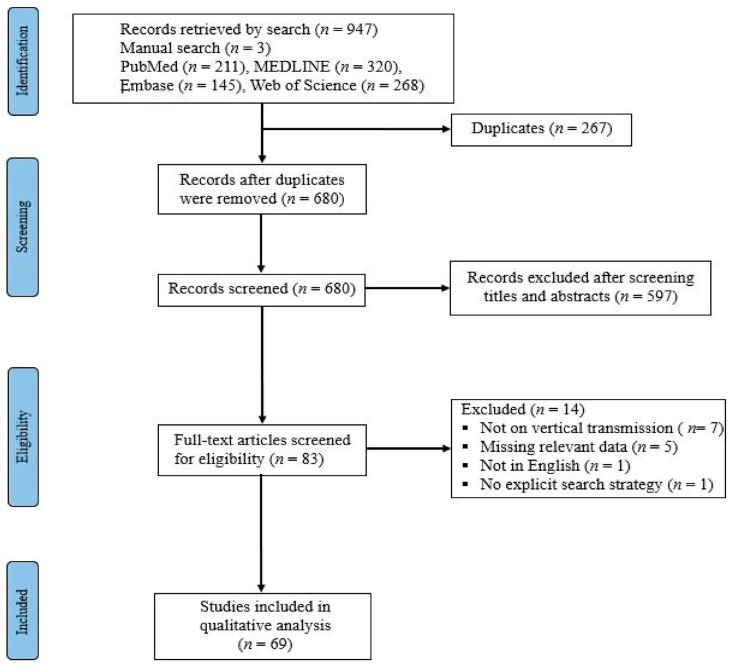
PRISMA diagram for the search and selection processes of the articles.

In total, 947 articles were identified through the electronic search of the databases (211, PubMed; 320, MEDLINE; 145, Embase; 268 Web of Science; and 3, from other sources). There were 680 studies left after duplicates were removed. After 597 articles were excluded by titles and abstracts screening, we retrieved 83 articles eligible for the full-text screening. We excluded 13 articles due to irrelevant or missing data on one or more variables of interest (based on the exclusion criteria mentioned earlier). Eventually, 69 studies that satisfied the eligibility criteria were included in this systematic review of systematic reviews for further synthesis and analysis. Figure 1 presents the flowchart of the study search and screening processes. The included studies were all systematic reviews on varieties of primary studies, with different study designs, including case reports, clinical practice guidelines, overviews, and observational studies. Overall, the included studies recruited over 54,413 pregnant women infected with SARS-CoV-2 consisting of more than 30,840 newborns delivered by the infected mothers. Among the neonates born from infected mothers, more than 800 neonates were reported as COVID-19 positive, out of which a majority of them likely acquired SARS-CoV-2 infection through environmental exposures, whereas a significant proportion likely acquired SARS-CoV-2 infection via vertical transmission. Table 1 presents the characteristics of the included studies.

### 3.2. Methodological Quality of the Included Studies

A majority of the included studies (92.75% (*n* = 64)) were of higher methodological quality, with 13.54% (*n* = 13) scoring the maximum obtainable score (18 points) on the quality-rating tool. All the included studies provided adequate details regarding “search method used to find evidence on the primary question(s) stated” (Item 1 on the OQAQ). Item 6 (were study quality assessment criteria used to inform the review analysis) scored the highest number for limited reporting (with 5 studies not reporting the details on the item and another 28 reporting the details partially). Avoidance of selection bias (Item 4) was partially reported in many of the included studies (48%, *n* = 33). The findings of the methodological quality are reported in Supplementary Materials Table S3.

### 3.3. SARS-CoV-2 Vertical Transmission

In the current systematic review, we identified 69 studies that satisfied all the inclusion criteria, reporting over 54,413 pregnancies infected with SARS-CoV-2 resulting in more than 30,840 newborns delivered by the infected mothers. Subsequently, the newborns were screened for COVID-19 using throat-swab SARS-CoV-2 RT-PCR testing or other standard diagnostic procedures for testing for COVID-19 infection from exposed individuals. Results from the samples tested revealed that there were over 800 neonates with positive SARS-CoV-2 results, indicating the plausibility of SARS-CoV-2 vertical transmission from COVID-19-infected mothers.

Furthermore, our analysis also showed that, although most of the reports during the early epidemic period suggested the plausibility of vertical transmission of SARS-CoV-2 [23,34,35,36,44,50,51,52,54,60,63,65,66,79,80,81,88], most of these reports hinted that more data were needed to confirm whether the mother-to-child transmission during pregnancy resulted from a vertical transmission or due to environmental exposure, such as during breastfeeding, hospital contamination by health workers, among others. Therefore, caution should be taken in making the conclusion that SARS-CoV-2 pathogens can be potentially transmitted vertically. In their systematic review, Smith et al. [23] identified one scenario of mother-to-child infection (neonatal infection) of COVID-19. They highlighted that, although SARS-CoV-2 could probably be transmitted vertically, there is still no concrete evidence to support the claim, likely due to the scarcity of the data at the moment. The neonatal infection could be during the post-delivery period, because the RT-PCR assay was performed when the infant was 36 h old. Moreover, none of the previous coronaviruses were reported to have been transmitted vertically [23]. However, that would not rule out the possibility of vertical transmission for SARS-CoV-2 [25].

Morevier, as the COVID-19 pandemic continues, many clinical and epidemiological features keep unfolding, and new knowledge is being discovered regarding SARS-CoV-2 transmission. Recently, several reports published in decent journals revealed that SARS-CoV-2 can be transmitted vertically and provided some clinical and epidemiological evidence to support the claim. Raschetti et al. [75] reported that the majority of mother-to-child infections (about 70%) of SARS-CoV-2 during pregnancy were likely transmitted due to environmental exposure (postpartum transmission). However, about 30% of the infections were likely due to vertical transmission, intrapartum or congenital. A reasonable number (i.e., about 9%) of the infections were confirmed to be potentially transmitted vertically.

### 3.4. Maternal and Newborn Death Rates

Maternal mortality was reported in 39 SRs, [16,17,18,20,21,22,23,25,34,35,36,39,40,41,46,47,51,52,54,55,57,60,62,63,64,65,67,68,72,73,74,76,77,78,79,80,87,88] and varied from 0% to 11.1% among the included reviews.

Newborns mortality was reported in 50 SRs, [16,17,18,19,20,21,22,23,25,34,35,36,39,40,41,43,45,46,47,49,50,51,52,53,54,56,57,59,60,62,63,64,65,67,68,69,70,71,73,74,76,77,78,79,80,82,84,85,87] and varied from 0% to 11.7% among the included reviews.

Our results are consistent with previous reports, including that of Vergara-Merino et al. [18]. Table 1 presents a summary of the results of maternal and newborns death rates.

### 3.5. Cesarean Delivery

A cesarean delivery (C-section) can be defined as a clinical surgical method used to deliver a an unborn child through incisions in the abdomen and uterus [89]. Our results show that a majority of the neonates who tested positive for SARS-CoV-2 were either delivered via C-section (cesarean delivery) [21], or had not been breastfed by the infected mothers [24]. For instance, according to Novoa et al. [21], there was more cesarean delivery (about 50.8%) than vaginal delivery (approximately 38.8%). Tolu et al. [24] reported that among the neonates with positive SARS-CoV-2 results, 54% were separated from their mother, and obviously, there was no breastfeeding during the separation period. Several reports also highlighted that high or low risk of mother-to-child transmission did not depend on the mode of delivery (i.e., either via C-section or virginal delivery) ([22,38,39,41,42], and the references therein).

## 4. Discussion

The connection between maternal with neonatal outcomes infected with SARS-CoV-2 during pregnancy has recently been reported and revealed some silent (and one of the most important) features of COVID-19. The transmission of COVID-19 directly from mother to child, or vertical transmission, has been an essential topic of discussion among researchers and public health experts, which raised concerns recently. Therefore, it needs urgent attention, from researchers and public health in order to protect an unborn vulnerable child from being infected with SARS-CoV-2. In this work, we conducted a systematic review of systematic reviews, based on available literature, focusing on the plausibility of vertical transmission of SARS-CoV-2 based on clinical evidence and scientific reports. Our results also included systematic review results that reported the issue of vertical transmission in pregnant women with other comorbid conditions, such as hypertensive disorders of pregnancy, maternal gestational diabetes, preterm birth (before the birth period of at least 37 weeks), labor induction, and post-partum hemorrhage.

Based on available evidence regarding SARS-CoV-2 transmission from mother to child through a placenta, we found that among all the 69 systematic reviews studies, most of them have hinted that vertical transmission of SARS-CoV-2 occurs postnatally due to environmental exposure during the pregnancy period. However, a significant percentage reported that SARS-CoV-2 could be transmitted vertically. Thus, our results highlighted that most early review reports suggested the plausibility of SARS-CoV-2 vertical transmission. Our findings are in line with previous reports [3,24,75]. In particular, Raschetti et al. [75] conducted a systematic review and meta-analysis based on 176 published cases of maternal outcomes infected with SARS-CoV-2. They found that 70% and 30% of neonates infected with SARS-CoV-2 were due to environmental exposures and vertical transmission, respectively. Moreover, according to more recent reports with availability of more relevant data or information on maternal perinatal and neonatal outcomes infected with SARS-CoV-2 during pregnancy, we found that SARS-CoV-2 can be potentially transmitted vertically. However, there is still a need for more adequate data to guide clinical recommendations with the required certainty of the evidence.

As SARS-CoV-2 continues to unfold, its biological system, especially in pregnant women infected with the disease, is still ambiguous. For vertical transmission to occur via the transplacental route, the SARS-CoV-2 infection should initially circle within the contaminated pregnant woman [90]. Previous works [90,91] reported that the virus attacks the uterine arterioles, then passes through the placenta’s fetal side, and reaches the chorionic villus and then continues to circulate in the fetus. Vis-a-vis catalytic feature, SARS-CoV-2 contamination can happen via enactment of the angiotensin-converting catalyst receptor on the outer layer of cells inside the fetus.

It is conjectured that SARS-CoV-2 vertical transmission during pregnancy through a transplacental route is likely to be higher when there is an increment in gestational age. This is thus thought to be added to the inexorably communicated angiotensin-converting protein 2 (ACE-2) receptors on the placenta nearer to the furthest limit of incubation [92]. Moreover, some proposed animal models highlighted that ACE-2 receptor articulation attains maximum, which is closer to the furthest limit of the gestational period [91,93]. In addition, some previous reports revealed that the identification of ACE-2 receptors on the human placenta differs amongst pregnant women. This could uphold the debate regarding why vertical transmission is uncommon and variable between COVID-19-infected pregnant women [94]. Fenizia et al. [12] reported that the two common receptors for SARS-CoV-2, that is, angiotensin-converting enzyme 2 (ACE2) and transmembrane protease serine 2, broadly spread in particular cell types of the maternal–fetal interface [12,91]. Thus, the effect of the SARS-CoV-2 on the placenta and the potential for vertical transmission need further investigation.

Furthermore, recent reports showed that the vertical transmission of COVID-19 during pregnancy likely occurs when there is a high viral burden and duplication level in the maternal blood [95]. However, SARS-CoV-2 viremia is uncommon amongst contaminated pregnant women. However, a few studies highlighted that a high viral load coupled with general inflammation could lead to viremia [96]. It has been recommended that there may likewise be a connection between the time/length of viral openness in utero and neonatal SARS-CoV-2 status. A lengthier size of viral openness might prompt increased probability of neonatal infection [91,97]. Nevertheless, solid proof is scarce for this issue, and future works are required to shed more light on mother-to-child transmission during pregnancy. Moreover, previous investigations divulged that various comorbidities might influence the probability of vertical transmission [91]. We also observed that the severity of disease progression did not increase the risk of vertical transmission [91,98].

Most of the previous works failed to address the issues bedeviling potential infection complications to both COVID-19-infected pregnant women and the outcomes. However, some included studies reported and discussed the plausibility of infection complications which could fuel the vertical transmission of SARS-CoV-2 during pregnancy. Based on previous studies, SARS-CoV-2 infection complications in pregnant women infected with COVID-19 and their newborns included maternal and neonatal mortality, preterm delivery, ruptured ectopic pregnancies, maternal depression, prematurity, abnormalities in amniotic fluid and the umbilical cord, placental abruption, gestational diabetes mellitus, fetal distress, abortion, vaginal bleeding, and fetal death (and stillbirth) [16,99]. These infection complications likely increase the possibility of the fact that there could be vertical transmission of SARS-CoV-2.

In summary, we conducted a systematic review of systematic reviews that provided evidence supporting the mother-to-child transmission of SARS-CoV-2 during pregnancy. We found that SARS-CoV-2 could be potentially transmitted vertically and could be likely due to the detection of SARS-CoV-2 RNA in placental tissue, which might lead to perinatal outcomes of infection with SARS-CoV-2. However, further investigation and analysis are needed, especially on amniotic fluid, a cord blood sample, and breast milk, to confirm/ascertain the possibility of SARS-CoCV-2 vertical transmission during pregnancy. Our results are consistent with recent studies [12,13,23], which revealed that, although evidence is still low, SARS-CoV-2 can be transmitted from a COVID-19-infected mother to the child via a vertical route.

Furthermore, several reports highlighted that most of the neonates infected with SARS-CoV-2 were delivered through a cesarean mode, indicating that the risk of vertical transmission does not solely depend on the delivery mode [21,24]. However, the long-term or short-term impact of SARS-CoV-2 vertical transmission should be investigated to protect vulnerable children from the adverse effect of SARS-CoV-2 mother-to-child infection during pregnancy. Also, more time is needed to follow up on the children infected with COVID-19 or other comorbid conditions of new babies born from infected mothers.

### 4.1. Strengths

The current systematic review study was conducted following an extensive literature search of well-known databases, including PubMed, MEDLINE, Embase, and Web of Science. Furthermore, relevant citations were extracted using the reference lists of the included studies to ensure robust coverage of the existing literature. The primary studies included a large number of pregnant women (in the first, second, and third trimesters of the pregnancy) and their diagnostic outcomes of SARS-CoV-2 for data from different countries across the globe. In addition, the justification for conducting the current study was the need to timely find and discuss the availability of the existing evidence on SARS-CoV-2 vertical transmission. It is also imperative to note that this study gathered all the relevant evidence available in the literature up to 7 July 2021, which is crucial in guiding public health authorities and policymakers on the association between maternal perinatal and neonatal outcomes infected with SARS-CoV-2 during pregnancy.

### 4.2. Limitations

The current study was not free from limitations. First, some of the included studies have poor quality, likely due to the potential risk of bias, small sample size, missing data, or lack of standard methodological approach. Secondly, heterogeneity among the included primary studies in terms of design could also serve a limiting factor. As such, interpretation of the findings should be done cautiously. Thirdly, due to the study period and the urgent need to investigate the issue of maternal perinatal with neonatal outcomes infected with SARS-CoV-2, and the fact that new reports related to COVID-19 are being published every day, we were unable to register our study with PROSPERO. However, as for the future, we plan to extend the current research when new information/relevant data become publicly available, which could help interpret the issue of vertical transmission of SARS-CoV-2 during pregnancy much better. We plan to have a proper registration of a review protocol before commencing the future update research. Furthermore, due to the inability of many previous works to address potential infection complications, which likely occurs to both COVID-19-infected mothers and their newborns, we did not provide an in-depth analysis regarding infection complications in the current work. Nevertheless, this issue would be given considerable effort in our future research. Therefore, it is imperative to emphasize that, as the COVID-19 pandemic is still in progress and many epidemiological features are unfolding, it is difficult to conclude how COVID-19 is transmitted vertically during pregnancy. Furthermore, it is plausible that some additional relevant information might have been published during this manuscript’s submission or publication process.

## 5. Conclusions

This systematic review of systematic reviews provides a general overview of maternal perinatal and neonatal outcomes infected with SARS-CoV-2. We found that SARS-CoV-2 could be potentially transmitted vertically. The results also indicated that the mode of delivery for women infected with SARS-CoV-2 did not increase or decrease the risk of COVID-19 infection for newborns but likely increased the risk for direct and indirect adverse health outcomes. Moreover, the maternal death rates were significantly higher in pregnant woman infected with COVID-19 than those without the disease [12,16]. However, prospective studies are needed to clarify the actual risk of SARS-CoV-2 mother-to-child infection and identify the optimal prevention and control strategies.

## Figures and Tables

**Table 1:** Features of the included studies.

Study	Database(s)	Date of Publication	Number of Included Studies	Number of Pregnant Women	Number of Newborns	Number of Infected Newborns	Maternal Deaths	Newborns Deaths	Cesarean Delivery	Remark (Vertical Transmission)	Sensitivity Analysis in Studies with Meta-Analysis
AbdelMassih et al. [19]	Embase, MEDLINE, Cochrane	April, 2021	64	1787	1787	45 (2.5)	NA	19	NA	Unlikely	Y
Abdollahpour et al. [33]	PubMed, Scopus, Embase, Science direct, ClinicalKey, PsycINFO, manual searches of other relevant papers	May, 2020	29	NA	2	NA	NA	NA	NA	No direct evidence	NM
Abou Ghayda et al. [16]	PubMed, Scopus, MEDLINE, Google Scholar, Embase	October, 2020	11	104	NA	NA	NA	6 (5.8)	52	Vertical infection was not found	NM
Akhtar et al. [20]	MEDLINE, PubMed, Scopus, Google Scholar	July, 2020	22	156	108	7	8	3	66	No evidence to support vertical transmission	NM
Allotey et al. [22]	MEDLINE, Embase, Cochrane database, WHO COVID-19 database, CNKI, Wanfang, L·OVE	September, 2020	77	11432	NA	NA	<1%	<1%	1060 (65% (57–73%) 95% CI)	Not reported	Y
Amaral et al. [34]	Embase, PubMed database	November, 2020	70	1457	1042	39 (3.7%)	15 (1%)	16 (1.5%)	15%	Low evidence to support vertical transmission	NM
Arroyo-Sanchez et al. [34]	MEDLINE/PubMed, ScienceDirect, ClinicalKey, LILACS, SciELO, Google Scholar, medRxiv, and SciELO Preprints.	2020	30	476	476	9 (1.9%)				Probable	NM
Ashraf et al. [35]	PubMed, Scopus, WoS, Embase, Google Scholar	July–September, 2020	21	90	92	4	1	1	81	Possible but rare	NM
Banaei et al. [36]	MEDLINE, Embase, Scopus, WoS, ProQuest, Google Scholar	2020	16	123	124	5	0	1	99	Probable	NM
Barcelos et al. [37]	PubMed, Scopus, Embase	Accepted November, 2020	27	NA	NA	9	NA	NA	NA	Possible, with low risk	NM
Bwire et al. [38]	PubMed/MEDLINE, Google Scholar	Accepted October, 2020	33	205	205	13 (6.3%)	NA	NA	71.9%	Low possibility of vertical transmission	NM
Cai et al. [39]	PubMed, Embase, Cochrane Library, Web of Science, Google Scholar, CBM	February, 2021	68	1019	1035	34 (3.29%)	14	6	59.71%	Possible	NM
Capobianco et al. [40]	PubMed, Scopus	Accepted July, 2020	13	114	108	5	0	2	95	Low probability of vertical transmission	N
Chamseddine et al. [41]	PubMed, medRxiv	October, 2020	48	245	201	6.45%	8 (3.2%)	2.5%	89%	Possible	NM
Chi et al. [42]	PubMed/MEDLINE, Embase, Cumulative Index to Nursing and Allied Health Literature, National Digital Library of Theses and Dissertations in Taiwan, Art Image Indexing Service on the Internet, Cochrane	February, 2021	14	107	105	8 (8.8%)	NA	NA	87.6%	Possible	NM
Della Gatta et al. [43]	PubMed, Scopus, CINAHL	July, 2020	6	51	48	1	NA	1	46	No evidence of vertical transmission	NM
Deniz & Tezer [44]	medRxiv, PubMed	July, 2020	50	714	606	20	NA	NA	NA	Potentially vertically transmit-ted	NM
Dhir et al. [45]	MEDLINE, Embase, Web of Science	August, 2020	86	1992	1141	58	NA	0	66%	Possible, with low risk	NM
Di Mascio et al. [46]	MEDLINE, Embase, CINAHL, ClinicalTrials.gov	May, 2020	6	41	42	0	0	2.4%	38	None has been reported	N
Di Toro et al. [25]	PubMed, Embase, medRxiv, Scholar, Scopus, Web of Science	January, 2021	24	588	444	19	5	3	85%	No evidence of vertical transmission	N
Diriba et al. [47]	PubMed, Web of Science, Embase, Google Scholar, Cochrane	September, 2020	23	1271	NA	0	1.5%	1.2%	57%	No evidence of vertical transmission	Y
Amaral et al. [34]	PubMed, Embase	November, 2020	70	1457	1112	39	15	16	597 (57.3%)	Possible	NM
Dube et al. [48]	PubMed, Embase, LitCovid, medRxiv, bioRxiv, Google Scholar, EBSCO MEDLINE, CINAHL, Scopus	November, 2020	72	NA	1408	51 (3.67%)	NA	NA	59.9%	Possible but low	NM
Dubey et al. [49]	PubMed	September, 2020	61	790	548	NA	NA	NA	72%	None reported	Y
Duran et al. [50]	Google Scholar, LILACS, PubMed	May, 2020	20	195	222	13	NA	1	48	Probable	NM
Elshafeey et al. [51]	LitCovid, EBSCO MEDLINE, CENTRAL, CINAHL, Web of Science, Scopus	May, 2020	33	385	256	4	1	1	175 (69.4)	Probable	NM
Galang et al. [52]	MEDLINE, ClinicalTrials.gov	August, 2020	31	98	94	8	1	1	72	Probable	NM
Gao et al. [53]	PubMed, Web of Science, Embase, MEDLINE	August, 2020	14	236	NA	1.8%	NA	1	69%	No evidence available	Y
Ghayda et al. [16]	PubMed, Scopus, MEDLINE, Google Scholar, Embase	October, 2020	11	104	NA	0	7	5 fetal + 1 neonatal	50%	No vertical transmission reported	N
Gordon et al. [54]	CINAHL, Embase, MEDLINEMEDLINE, PubMed	May, 2020	8	NA	46	7	NA	0	86%	Likely	NM
Han et al. [55]	MEDLINE, PubMed, Web of Science, Cochrane, CNKI, Wanfang Data, VIP, SinoMed, ClinicalTrials.gov	October, 2020	36	1103	NA	<0.01%	<0.01%	<0.01%	28.59%	Unlikely	NM
Hassanipour et al. [56]	PubMed, Embase, Scopus, Web of Science, Google Scholar	December, 2020	10	135	NA	1	NA	1	84%	Low evidence for vertical transmission	N
Huntley et al. [57]	MEDLINE, Ovid, ClinicalTrials.gov, medRxiv, Scopus	August, 2020	13	538	435	0	0	1	85%	None reported	NM
Islam et al. [58]	PubMed, Embase, Scopus, Google Scholar, Web of Science	December, 2020	13	235	NA	0	NA	NA	156 (66.38%)	Not reported	N
Jafari et al. [59]	Embase, Scopus, PubMed, Web of Science, Cochrane	January, 2021	121	NA	8%	NA	NA	2.5%	48%	Possible	N
Juan et al. [60]	PubMed, Embase, Cochrane, CNKI, Wanfang Data	May, 2020	24	324	240	3	7	1	78.1%	Possible	NM
Karabay et al. [61]	MEDLINE, Web of Science, PubMed, ScienceDirect, CINAHL, Scopus, Cochrane, TUBiTAK	November, 2020	35	NA	NA	68	NA	0	NA	Possible	NM
Kasraeian et al. [62]	PubMed, Google Scholar, medRxiv, UpToDate search engines	May, 2020	9	87	86	0	0	0.2%	92.2%	Not reported	Y
Khalil et al. [63]	MEDLINE, Embase, ClinicalTrials.gov, Cochrane	August, 2020	86	2567	NA	1.4%	0.9%	0.6%	48.3%	Possible	N
Kotlyar et al. [13]	PubMed, Embase, medRxiv, bioRxiv	Jan, 2021	69	1566	936	27 (3.2%)	NA	NA	73%	Possible	Y
Lopes de Sousa et al. [64]	PubMed, Scopus, Embase, ScienceDirect, WoS, Google Scholar, bioRxiv, medRxiv	June, 2020	49	755	598	1.8%	8	10	64.7%	No concrete evidence of vertical transmission	NM
Matar et al. [65]	Ovid MEDLINE and Epub Ahead of Print, In-Process and Other Nonindexed Citations, Ovid Embase, Ovid Cochrane Central Register of Controlled Trials, Scopus	June, 2020	24	136	94	2	1	3	76.3%	Probable	N
Melo et al. [66]	PubMed, Scopus, LILACS, Web of Science, Google Scholar, Preprints, bioRxiv, medRxiv	July, 2020	38	520	NA	16	NA	NA	NA	Possible	Y
Mirbeyk et al. [67]	PubMed, Web of Science, Google Scholar, Scopus, WHO COVID-19 database	April, 2021	37	386	302	5%	2	3	86%	Possible, with low evidence	NM
Muhidin et al. [68]	PubMed, Scopus, Embase, ProQuest, ScienceDirect	April, 2020	9	89	89	0	0	2.2%	91.9%	No evidence	NM
Najafi et al. [69]	PubMed, Scopus, Google Scholar	December, 2020	20	NA	145	7	NA	10%	NA	Low risk of vertical transmission	NM
Neef et al. [70]	PubMed, Google Scholar, Web of Science	October, 2020	32	258	261	12	NA	3	NA	The risk of vertical transmission is low	N
Novoa et al. [21]	MEDLINE, Embase, Cochrane Library, LILACS, CNKI, VIP, Wanfang Data	January–February, 2021	37	322	195	16	1	1	99 (50.8%)	No concrete evidence to support vertical transmission	N
Oltean et al. [71]	MEDLINE, Embase, Google Scholar, WHO database on COVID-19, Disaster Lit: Database, medRxiv, and OSF Preprints	March, 2021	41	315	262	3.1%	NA	1	NA	Vertical transmission has yet to be confirmed	NM
Oshay et al. [72]	PubMed, Embase, World Health Organization, Google Scholar	July, 2021	67	427	304	3.2%	8	NA	NA	Low risk of vertical transmission	NM
Papapanou et al. [17]	PubMed, Scopus, Cochrane	January, 2021	39	NA	NA	1.6–10%	<2%	<3%	52.3–95.8%	Probable	NM
Pastick et al. [73]	PubMed/MEDLINE	August, 2020	126	11308	10597	NA	33	0.7%	70%	Not reported	NM
Pettirosso et al. [74]	MEDLINE, Embase, WHO COVID-19 database, Cochrane	August, 2020	60	1287	NA	19	8	6	NA	Possible	NM
Raschetti et al. [75]	PubMed, Cochrane, Web of Science, bioRxiv, and medRxiv	October, 2020	74	NA	NA	53	NA	NA	NA	Confirmed/possible	N
Rodrigues et al. [76]	PubMed, Scopus, Web of Science, medRxiv	November, 2020	161	3985	2015	61	28	10	53.92%	Possible	NM
Smith et al. [23]	PubMed, MEDLINE, Embase	June, 2020	9	92	60	1	0	1	80%	Probable	NM
Thomas et al. [77]	Ovid MEDLINE, Embase, CENTRAL	July, 2020	18	157	160	5	1	1	115 (73%)	No conclusive evidence to support vertical transmission	NM
Tolu et al. [24]	PubMed, CINAHL, Web of Science, Scopus, CENTRAL	April, 2021	51	NA	336	15	NA	NA	265 (78.8%)	Not enough evidence on vertical transmission	NM
Trippella et al. [78]	MEDLINE, Embase, Google Scholar, medRxiv	June, 2020	37	275	248	16	1	1	74.9%	Possible	NM
Trocado et al. [79]	PubMed, Scopus database, and WHO database	July, 2020	8	95	51	1	0	1	94%	Probable	NM
Turan et al. [80]	PubMed, Ovid MEDLINE, WoS, China Academic Literature Database	July, 2020	63	637	479	8	10	5	83%	Probable	NM
Vergara-Merino et al. [18]	PubMed/MEDLINE, Embase, other electronic databases, clinical trials registries, and preprint repositories, among other sources relevant to COVID-19	February, 2021	52	NA	NA	0–11.5%	0–11.1%	0–11.7%	48.3–100%	Possible	NM
Walker et al. [81]	MEDLINE, Embase, Maternity and Infant Care Database	June, 2020	49	655	666	28	NA	NA	5.3%	Probable	NM
Yang et al. (a) [82]	PubMed, Google Scholar, CNKI, Wanfang Data, VIP, CBMdisc	April, 2020	18	114	NA	7	NA	1	90.8%	No direct evidence	NM
Yang et al. (b) [83]	PubMed, CNKI, CBMdisc, Wanfang Data	2020	22	NA	83	9	NA	NA	88%	No direct evidence	NM
Yee et al. [84]	PubMed, Embase, WoS	October, 2020	9	93	103	4	NA	0	NA	No direct evidence	N
Yoon et al. [85]	PubMed/MEDLINE, Embase	2020	28	223	201	4	NA	1	88.1%	Probable	NM
Yuan et al. [86]	PubMed, MEDLINE, CBM, Wanfang	2021	29	564	555	18	NA	NA	62.8%	No sufficient evidence to exclude the possibility of vertical transmission	NM
Zaigham et al. [87]	MEDLINE, Embase, Google Scholar	April, 2020	18	108	87	1	0	1	92%	Probable	NM

Abbreviation: NA: Not available; Remark: Conclusion remarks drawn from the included systematic reviews studies on vertical transmission of SARS-CoV-2; Y: Yes; N: No; NM: No Meta-analysis.

## Data Availability

All data used in this work were publicly available.

## Supplementary Materials

The following are available online at https://www.mdpi.com/article/10.3390/v13091877/s1, Table S1: Search Strategy, Table S2: OQAQ: Overview Quality Assessment Tool for Systematic Reviews and Meta-Analyses, Table S3: Quality rating using OQAQ.
