# Supplementary material for: Vertical Transmission of SARS-CoV-2: A Systematic Review of Systematic Reviews
Source: Viruses. 2021 Sep 20;13(9):1877. doi: 10.3390/v13091877 (PMC8471858; doi:10.3390/v13091877)
Supplement: Supplementary file 1 [file viruses-13-01877-s001.zip › viruses-1369611-supplementary.pdf]

viruses

MDPI

Supplementary materials

Table S1. Search strategy.We searched PubMed, MEDLINE, Embase, and Web of Science in accordance with PRISMA guidelines, from December 1, 2019, to July 7, 2021, for published review papers that reported the issue of vertical transmission of SARS-CoV-2 (mother-to-child transmission of COVID-19) during pregnancy period. We searched all fields for coronavirus (search terms: "COVID-19", "SARS-CoV-2"), terms of vertical transmission and pregnancy (search terms: "antenatal", "prenatal", "vertical transmission", "pregnancy", "pregnant", "delivery", "infant", "neonate", "newborn"), and terms of reviews (search terms: "systematic review*").Full search strategies for each database are given in Table S1.

Table S1. Search strategy.

Database	Number of Studies	Search Terms
PubMed	211	("COVID-19" OR "SARS-CoV-2") AND ("antenatal" OR "prenatal" OR "vertical transmission" OR "pregnancy" OR "pregnant" OR "delivery" OR "infant" OR "neonate" OR "newborn") AND ("systematic review*")
Medline	320	("COVID-19" OR "SARS-CoV-2") AND ("antenatal" OR "prenatal" OR "vertical transmission" OR "pregnancy" OR "pregnant" OR "delivery" OR "infant" OR "neonate" OR "newborn") AND ("systematic review*")
Embase	145	('COVID-19' OR 'SARS-CoV-2') AND ('antenatal' OR 'prenatal' OR 'vertical transmission' OR 'pregnancy' OR 'pregnant' OR 'delivery' OR 'infant' OR 'neonate' OR 'newborn') AND ('systematic review*')
Web of Science	268	((TS=COVID-19) OR (TS=SARS-CoV-2)) AND ((TS=antenatal) OR (TS=prenatal) OR (TS=vertical transmission) OR (TS=pregnancy) OR (TS=pregnant) OR (TS=delivery) OR (TS=infant) OR (TS=neonate) OR (TS=newborn)) AND ((TS=systematic review*))

Table S2. OQAQ: Overview Quality Assessment Tool for Systematic Reviews and Meta-Analyses.

**1. Were the search methods used to find evidence on the primary question(s) stated?**
(a) Yes, description of databases searched, search strategy, and years reviewed. 2 points.<br>(b) Partially, descriptions of methods not complete. 1 point.<br>(c) No, no description of search methods. 0 points.
2. Was the search for evidence reasonably comprehensive?
(a) Yes, at least one computerised database searched and also a search of unpublished or non-indexed literature. 2 points.<br>(b) Can't tell, search strategy partially comprehensive, at least one of the strategies performed. 1 point.<br>(c) No, search not comprehensive or not described well. 0 points.
3. Were the criteria used for deciding which studies to include in the review reported?
(a) Yes, inclusion and exclusion criteria clearly defined. 2 points.<br>(b) Partially, reference to inclusion and exclusion criteria can be found but are not defined clearly enough. 1 point.<br>(c) No, no criteria defined. 0 points
4. Was bias in the selection of articles avoided?
(a) Yes, issues influencing selection bias were covered. Both of the following bias-avoiding strategies were used: (1) two or more assessors independently judged study relevance, (2) assessors selected studies using predetermined criteria. 2 points.<br>(b) Can't tell, only one of the strategies used. 1 point.

(c) No, selection bias was not avoided or was not discussed. 0 points.
5. Were the criteria used for assessing the methodological quality of studies reviewed reported?
(a) Yes, criteria defined and used addressed the major factors influencing bias. 2 points.
(b) Partially, some discussion or reference to criteria. 1 point.
(c) No, validity or methodological quality criteria not used or not described. 0 points.
6. Were study quality assessment criteria used to inform the review analysis?
(a) Yes, criteria were used to inform the analysis, either by exclusion from the analysis if low quality or through sensitivity analysis. 2 points.
(b) Partially, some discussion but not clearly described application of criteria. 1 point.
(c) No, criteria not used or not described. 0 points.
7. Were the methods used to combine the findings of the relevant studies (to reach a conclusion) reported?
(a) Yes, qualitative and quantitative methods are acceptable. 2 points.
(b) Partially, partial description of methods to combine and tabulate; not sufficient to duplicate. 1 point.
(c) No, methods not stated or described. 0 points.
8. Were findings of the relevant studies combined appropriately relative to the primary question of the overview?
(a) Yes, combining of studies appears acceptable. 2 points.
(b) Can't tell, should be marked if in doubt. 1 point.
(c) No, no attempt was made to combine findings, and no statement was made regarding the inappropriateness of combining findings. 0 points.
9. Were the conclusions made by the author(s) supported by the data and/or analysis reported in the overview?
(a) Yes, data were reported that support the main conclusions regarding the primary question(s) that the overview addresses. 2 points.
(b) Partially. 1 point.
(c) No, conclusions not supported or unclear. 0 points.

Table S3. Quality rating using OQAQ.

**Study**	**OQAQ Score**									
	**Qu 1**	**Qu 2**	**Qu 3**	**Qu 4**	**Qu 5**	**Qu 6**	**Qu 7**	**Qu 8**	Qu 9	**Total**
AbdelMassih et al. 2021	2	2	2	2	2	**2**	2	2	2	18
Abdollahpour et al. 2020	2	2	2	1	1	0	2	2	2	14
Abou Ghayda et al. 2020	2	1	2	1	2	2	2	2	2	16
Akhtar et al. 2020	2	2	2	2	2	2	2	2	2	18
Allotey et al. 2020	2	2	2	2	2	2	2	2	2	18
Amaral et al. 2020	2	2	2	2	2	1	2	2	2	17
Arroyo-Sanchez et al. 2020	2	1	1	1	1	0	2	2	1	11
Ashraf et al. 2020	2	2	1	2	2	1	2	2	1	15
Banaei et al. 2020	2	2	2	1	2	1	2	2	2	16
Barcelos et al. 2021	2	1	1	1	2	1	2	2	1	13
Bwire et al. 2020	2	2	1	1	2	2	2	2	2	16
Cai et al. 2021	2	2	2	2	2	2	2	2	2	18
Capobianco et al. 2020	2	2	2	2	2	2	2	2	2	18
Chamseddine et al. 2020	2	2	1	1	2	1	2	2	2	15
Chi et al. 2021	2	2	2	1	2	1	2	2	2	16
Della Gatta et al. 2020	2	1	2	1	2	1	2	2	2	15
Deniz & Tezer 2020	2	2	1	1	2	1	2	2	2	15
Dhir et al. 2020	2	2	2	1	2	1	2	2	2	16
Di Mascio et al. 2020	2	2	2	1	2	2	2	2	2	17
Di Toro et al. 2021	2	2	2	2	2	2	2	2	2	18
Diriba et al. 2020	2	2	2	2	2	2	2	2	2	18
do Amaral et al. 2020	2	2	2	1	2	2	2	2	2	17

Dube et al. 2020	2	2	2	1	2	1	2	2	2	16
Dubey et al. 2020	2	1	2	2	2	2	2	2	2	17
Duran et al. 2020	2	2	1	1	2	1	2	2	2	15
Elshafeey et al. 2020	2	2	1	1	1	1	2	1	1	12
Galang et al. 2020	2	1	1	1	1	0	1	2	2	11
Gao et al. 2020	2	2	2	2	2	2	2	2	1	17
Ghayda et al. 2020	2	1	2	1	2	1	2	2	2	15
Gordon et al. 2020	2	2	2	2	2	2	2	2	1	17
Han et al. 2020	2	2	2	1	2	1	2	2	2	16
Hassanipour et al. 2020	2	2	2	2	2	2	2	1	2	17
Huntley et al. 2020	2	2	2	1	2	1	2	1	2	15
Islam et al. 2020	2	2	2	1	2	1	2	1	1	14
Jafari et al. 2021	2	2	2	2	2	2	2	2	2	18
Juan et al. 2020	2	2	2	1	2	2	2	2	2	17
Karabay et al. 2020	2	1	2	2	2	2	2	2	2	17
Kasraeian et al. 2020	2	1	1	2	2	2	2	2	1	15
Khalil et al. 2020	2	2	2	2	2	2	2	2	1	17
Kotlyar et al. 2021	2	2	2	2	2	2	2	2	2	18
Lopes de Sousa et al. 2020	2	2	2	1	2	1	2	2	2	16
Matar et al. 2020	2	2	2	2	2	2	2	2	2	18
Melo et al. 2020	2	2	2	2	2	2	2	1	2	17
Mirbeyk et al. 2021	2	1	1	1	1	1	2	2	2	13
Muhidin et al. 2020	2	2	2	2	2	2	2	2	1	17
Najafi et al. 2020	2	2	2	2	2	1	2	2	2	17
Neef et al. 2020	2	1	2	2	2	2	2	2	2	17
Novoa et al. 2021	2	1	2	2	2	2	2	2	2	17
Oltean et al. 2021	2	2	2	1	2	1	2	2	2	16
Oshay et al. 2021	2	2	2	2	2	2	2	2	2	18
Papapanou et al. 2021	2	2	2	2	2	1	2	2	2	17
Pastick et al. 2020	2	1	1	1	2	1	2	2	2	14
Pettirosso et al. 2020	2	1	2	1	2	1	2	2	2	15
Raschetti et al. 2020	2	2	2	2	2	2	2	2	2	18
Rodrigues et al. 2020	2	1	2	1	2	1	2	2	2	15
Smith et al. 2020	2	2	2	2	2	2	2	2	1	17
Thomas et al. 2020	2	2	2	1	2	1	2	2	1	15
Tolu et al. 2021	2	2	2	1	2	1	2	2	2	16
Trippella et al. 2020	2	2	2	1	2	2	2	2	2	17
Trocado et al. 2020	2	1	2	2	2	1	2	2	1	15
Turan et al. 2020	2	2	1	2	2	2	2	2	2	17
Vergara-Merino et al. 2021	2	2	2	2	2	2	2	2	2	18
Walker et al. 2020	2	1	2	2	2	0	2	2	2	15
Yang et al. (a) 2020	2	1	2	2	2	0	2	2	1	14
Yang et al. (b) 2020	2	1	1	2	2	1	2	2	2	15
Yee et al. 2020	2	1	2	2	2	2	2	2	1	16
Yoon et al. 2020	2	2	2	1	2	2	2	2	2	17
Yuan et al. 2020	2	1	2	2	2	2	2	2	2	17
Zaigham et al. 2020	2	1	2	1	2	2	2	2	1	15

Abbreviations: QU, question.

OQAQ Questions:

1. Were the search methods used to find evidence on the primary question(s) stated?
2. Was the search for evidence reasonably comprehensive?
3. Were the criteria used for deciding which studies to include in the review reported?
4. Was bias in the selection of articles avoided?
5. Were the criteria used for assessing the methodological quality of studies reviewed reported?
6. Were study quality assessment criteria used to inform the review analysis?
7. Were the methods used to combine the findings of the relevant studies (to reach a conclusion) reported?
8. Were findings of the relevant studies combined appropriately relative to the primary question of the overview?
9. Were the conclusions made by the author(s) supported by the data and/or analysis reported in the overview?
